# Is standard breast-conserving therapy (BCT) in elderly breast cancer patients justified? A prospective measurement of acute toxicity according CTC-classification

**DOI:** 10.1186/1748-717X-5-103

**Published:** 2010-11-04

**Authors:** Razvan M Galalae, Jürgen Schultze, Kirsten Eilf, Bernhard Kimmig

**Affiliations:** 1 Paul Scherrer Institute, Villigen PSI, Switzerland; 2 Medical Faculty, Christian-Albrechts-University, Kiel, Germany; 3 Clinic for Radiation Therapy (Radiooncology), University Hospital Schleswig-Holstein, Campus Kiel, Germany

## Abstract

**Background:**

Breast conserving therapy (BCT) is an accepted treatment for early-stage breast cancer. This study aimed to measure prospectively acute radiation-related toxicity and to create a comprehensive data base for long-term temporal analyses of 3D conformal adjuvant radiotherapy. The specific aspect of age has been neglected by traditional research. Therefore, the impact of age on acute BCT toxicity should be also specifically adressed.

**Methods:**

Toxicity was measured in 109 patients at initiation (t1), during radiotherapy (t2-t7), and 6 weeks after treatment completion (t8) using a new topographic module. Organ systems were recorded in 15 scales and scored according to symptom intensity (grade 0-5) based on CTC (Common Toxicity Criteria) -classification. Radiotherapy was virtually CT-based planned and applied with 6-MeV-photons. Mean total dose was 60.1 Gy. Patients were stratified by age in 3 Groups: <50, 50-60, and >60 years.

**Results:**

Registered toxicity was generally low. Mean overall-grade climbed from 0.29-0.40 (t1-t7), and dropped to 0.23 (t8). Univariate analyses revealed slightly higher toxicity in older (> 60 years) versus young patients (< 50 years) in 2 scales only: breast-symmetry (p = 0.033), and arm function (p = 0.007). However, in the scale "appetite" toxicity was higher in younger (< 50 years) versus older (> 60 years) patients (p = 0.039). Toxicity differences in all other scales were not significant. Between older (> 60 years) and midaged patients (50-60 years) no significant differences in toxicity were found. This was also true for the comparison between young (< 50 years) versus midaged patient groups (50-60 years).

**Conclusion:**

The treatment concept of BCT for breast cancer is generally well tolerated. The toxicity-measurement with the new topographic module is feasible. Not modified standard treatment for BC should be performed in elderly women.

## Introduction

With the aging of the population, more older women are being diagnosed with breast cancer. Over 40% of all newly diagnosed breast cancer cases in the United States occur in the age subgroup of postmenopausal women and only 5% to 7% of breast carcinomas are diagnosed in women who are younger than 40 years of age [1]. Higher mortality in younger breast cancer population was attributed in previous studies to poorer outcomes in early-stage disease [2-4]. Although elderly women do at least as well as younger patients in survival time for localized and regional stages of breast cancer, therapy-related adverse effects and initially impaired general health condition can influence the older individual's functional health status in cancer survivors. A view on this interplay and clinical dilemma might be reflected in the tendency of undertreatment and/or non-standard therapy in older breast cancer population. However, Sweeney et al. [5] provided data of well functioning 2218 female long-term cancer survivors, when compared with 23501 women without a cancer history (patients aged 55 to 69 years). They also found that patients who were less than 2-year cancer survivors had a higher prevalence of limitations than women who were survivors of 2 or more years. Thus, recovery from effects of the disease and its treatment would take place over time. This is consistent with other reports [6]. These results emphasize the need to focus on elderly women for screening, early detection, diagnostic evaluation, and therapy but in a more comprehensive way by analyzing prospectively temporal variations of outcomes in comparison with a pre-therapeutic first assessment. This is especially true for acute toxicity which is generally neglected by traditional research. Therefore, knowledge on frequency and severity of acute breast cancer therapy-related morbidity is very limited.

Breast-conserving therapy (BCT) is the accepted standard treatment for early-stage breast cancer (BC) and consists of conserving surgery (CS) and postoperative adjuvant radiotherapy (RT) [7,8]. The current study aimed to measure prospectively the acute treatment toxicity in general and to create a comprehensive data base for lomg-term temporal analyses of 3D conformal adjuvant radiotherapy, which was developed throughout the late 1990 s in order to lower radiotherapy-related effects of BCT. The special aspects of age and its impact on acute BCT-related toxicity should be also specifically addressed.

## Patients and methods

### Study design and instruments

Therapy-related toxicity is an independent and important endpoint of modern treatment concepts [9]. According to the Radiation Therapy Oncology Group (RTOG) classification, treatment-related normal tissue reactions between day 0 and 90 following radiotherapy initiation are labeled "acute". The National Cancer Institute (NCI) developed the original Common Toxicity Criteria (CTC) in 1982 in an effort to provide standard language for reporting adverse events occurring in cancer clinical trials. In the current study, the CTC criteria [10] constituted the basis of the therapy-related morbidity documentation. The acute adverse events were scored in six categories from grade 0 (no events), grade 1 (mild event), grade 2 (moderate event), grade 3 (severe event), grade 4 (life-threatening event), to grade 5 (death related to adverse event). All patients consecutively treated in the study time were selected for enrolment in order to avoid bias by defining exclusion criteria (e.g. incomplete treatment). Main aim of the study was to create a pre-irradiation data base for prospective outcome analyses in order to evaluate temporal outcome trends of the introduced 3D conformal adjuvant radiotherapy within the BCT. In phase 1 (the present study report), the acute radiation-related toxicity and the specific aspects of age shoud be first adressed in a patient sample of n = 100. Phase 2 (subject of later report) should adress the treated patients at long-term follow-up in comparisson with the registred pre-therapeutic and acute morbidity. This study was an important part of a broad and comprehensive department effort to create a multi-entity electronic research data base [11]. To this end, documentation instruments based on the CTC classification were developed for several anatomic- topographic body regions: central nervous system, head & neck, breast, thorax, abdomen/pelvis [12]. These topographic modules aimed to assess with standardized and organ system-related operability acute radiation-caused adverse events facilitating interdisciplinary comparisons. Total radiation treatment time for breast cancer lasts 6 weeks. A follow-up visit 6 weeks after completion of radiotherapy is necessary to complete the acute phase - the first three months following treatment initiation. Therefore, the study design envisaged the prospective toxicity measurement at initiation (t1), during treatment (t2-t7), and 6 weeks after radiotherapy completion (t8) using the new developed topographic module. When age factors are presented in clinical studies for breast cancer, they are usually reported according to the age break of < 50 and 50 years or more to approximate those differences imposed by menopause [13]. This cut-off level was also used in the present study to define young age, 50 to 60 years for midage [14], as well as > 60 years for older population.

### Computed tomography (CT)-based virtual radiotherapy planning, target volumes and applied dose

**Figure 1:**
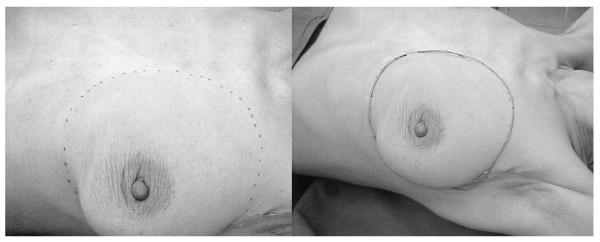
**Clinical assessment of breast anatomy and marking**.

**Figure 2:**
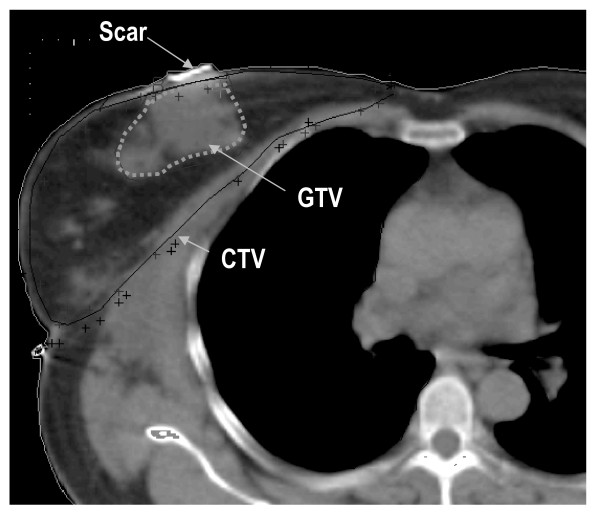
**Target volume definition on CT**.

The technology chain for CT-based virtual simulation consisted in a CT-scanner, a virtual simulator, a networking system, and a 3D radiotherapy planning system. Modern linear accelerators were used for radiation application. Interconnectivity between the various equipment used was provided by the standard data format - Digital Imaging and Communications in Medicine-Radiotherapy (DICOM RT) [15]. In the planning process a CT-study was first performed. Patients were immobilized using standardized devices. Breast anatomy was assessed by palpation and marked with radioopaque wires on skin, which was important to discriminate in CT images between breast and fatty tissue (figure 1). The first reference point was positioned in the scanned area using a laser system and highlighted with radioopaque markers. The CT study was then performed with a slice collimation of 5 mm and exported to the virtual simulator, where the target contouring occurred. According ICRU (International Commission of Radiation Units and Measurements) Report 50 [16] the Gross Tumor Volume (GTV) was defined in the region of the tumor bed after breast-conserving surgery and was expanded to the entire breast to account for subclinical disease (Clinical Target Volume, CTV). Organs at risk were the lung and the contralateral breast. In nodal negative breast cancer patients the axillar region was defined as organ at risk as well. In addition, in left-sided tumor lesions the heart was also taken into consideration and in right-sided tumor sites the liver. Target contouring was performed digitally in all transversal CT-slices. Figure 2 shows the GTV and the CTV in one CT cross-section. The scar was also radioopaque marked. In the third planning step, the CT study with digitized target volumes was exported to the radiotherapy planning system. In Observer-Eye-View (OEV) beam incidence, geometry and size were determined. Each beam was then conformed in Beam-Eye-View (BEV) perspective to the defined target using a remote multi-leave-collimator (MLC) of a linear accelerator. A small safety margin was added to form the Planning Target Volume (PTV). A dose distribution as homogeneous as possible was than calculated. Nodal negative patients were treated by a tangential two-field-technique to a dose of 50 Gy. In nodal positive patients the supraclavicular region was additionally irradiated to a dose of 46 Gy using an asymmetric three-field-technique. In cases with advanced axillar tumor involvement or lymph node capsule penetration the axillar region was added to the target and treated to a dose of 46 to 50 Gy. Fractionation was conventional with 2 Gy daily. Six MeV photons from an accelerator were used. The GTV was boosted to the total dose of 60 to 64 Gy depending on the surgical margin and using fast electrons from an accelerator. The mean applied total dose was 60.1 Gy.

### Patient and tumour characteristics

Hundred-nine consecutively treated patients with breast cancer were prospectively analysed. Mean age was 55 years (26-81 years). Fourteen patients (12.9%) were younger than 50 years, 58 (53.2%) were aged between 50 and 60 years, and 37 (33.9%) were older than 60 years. All other tumour characteristics including stage distribution [17] are detailed in table 1. Pathology reports revealed in 80.7% invasiv ductal and in 9.2% invasiv lobular carcinoma; in 3.7% a DCIS was found. Other entities were summarized in the remaining cohort.

**Table 1:** Tumor characteristics

Variable	N	Valid Percentage
**T-Stage** **according UICC**		
pT1	66	60.6
pT2	38	34.8
pT3	1	0.9
Tis	4	3.7
		
**N-Stage** **according UICC**		
pN0+cN0	68+3	65.1
pN1a	7	6.4
pN1bi	12	11.0
pN1bii	5	4.6
pN1biii	11	10.1
pN1biv	2	1.8
pN2	1	0.9
		
**Grading**		
G1	6	5.5
G2	55	50.5
G3	34	31.2
Unknown	14	12.8
		
**Total**	109	100

Performed surgical modality was in 86.2% a segmentectomy, in 8.3% a lumpectomy, and in 3.7% a quadrantectomy. In two cases a mastectomy with expander reconstruction was performed. Mean minimal resection margin was 0.5 cm (range 0 to 2 cm). Axillary lymph node dissection was performed in 97.2%, in 20.2% by using the sentinel lymph node biopsy method. Mean number of removed lymph nodes was 16.5 (0-29). In three DCIS-patients an axillary lymph node dissection was not performed. Postoperative complications after breast cancer surgery (e. g. mastitis, thrombophlebitis, wound complications) were generally not severe and occurred in 31.2% of the patients; 68.8% were complication-free. Seroma development was the most frequent postoperative complication in 11.9%. Estrogen receptor status was in 64.2% positive, mean score was 6 (range 1-12). In 35.8% estrogen receptor status was negative. Progesterone receptor status was in 57.8% positive, mean score was 4.9 (range 1-12). In 42.2% progesterone receptor status was negative.

### Adjuvant therapy, radiotherapy techniques

Adjuvant systemic treatment was carried out in patients with one or more poor prognostic factors (high T-stage, lymph node involvement, high grading, negative estrogen and/or progesterone receptor status), and initiated following surgery. Forty-one patients were treated with adjuvant chemotherapy: in 31 patients with CMF (cyclophosphamide/methotrexate/5-FU)-chemotherapy and all other patients with EC (epirubicin/cyclophosphamide). Forty-five patients were treated by adjuvant hormonal therapy with tamoxifen.

Seventy-one (65.1%) patients were irradiated with a tangential 2-field-technique (breast only), and 38 (34.8%) locoregionally with an asymmetric 3-field-technique (24 supraclavicular region only, and 14 supraclavicular/axillar region). The median total dose was 60 Gy (GTV). The median dose in the breast was 50 Gy, and in the axilla and/or the supraclavicular region it was 46 Gy.

### Assessment of therapy-related toxicity

Toxicity was measured using a newly developed topographic module. The instrument contained six sections: skin, breast, axilla, arm, general symptoms, and impairments by therapy. Those sections were subdivided in 15 scales: skin 2 scales, breast 3 scales, axilla 3 scales, arm 2 scales, other symptoms 1 scale, general symptoms 3 scales, and impairment by radiation 1 scale. All scales were scored according to symptom intensity of CTC-classification in maximal 5 severity grades from grade 0 (no events) to grade 5 (death related to adverse events). The skin was evaluated in the scales "pigmentation" and "dermatitis". Breast was evaluated according "symmetry", "lymphedema", and "pain". Axillary toxicity was scored in "pain", "hair loss", "sweat gland function". For the ipsilateral arm "lymphedema", and "function/mobility" were assessed. General symptoms (appetite, nausea, and Karnofsky [18] index) were also rated. The scale "impairments by radiotherapy" judged (actively requested by the physician) overall difficulties from patient's perspective caused by therapy in 5 severity grades as well. Data were analysed using the Statistical Package for the Social Sciences (SPSS) for Windows.

## Results

Mean grades of symptom severity were calculated for all scales during the acute phase of radiotherapy (t1 to t8). Toxicity was generally very low. The mean grade did not exceed the maximum of 1.057 (with a possible range from 0 to 5). Clinically relevant grade 3-toxicity was extremely low and seen in three scales only: "skin dermatitis", "breast symmetry", and "breast lymphedema" (table 2). Severe toxicity ≥ grade 4 was not observed. In all other scales only mild to moderate or no events events (grade 0-2) were registered.

**Table 2:** Toxicity documentation: measurement points t1-t8 (Percentages)

Scale	Mesurement-point	Grade 0	Grade 1	Grade 2	Grade 3	Grade 4
**Skin: Dermatitis**	**t1**	91.01%	6.74%	1.12%	**1.12%**	0.00%
	**t2**	86.96%	11.96%	1.09%	0.00%	0.00%
	**t3**	75.00%	22.83%	2.17%	0.00%	0.00%
	**t4**	58.06%	37.63%	3.23%	**1.08%**	0.00%
	**t5**	33.70%	47.83%	17.39%	**1.09%**	0.00%
	**t6**	28.57%	51.65%	19.78%	0.00%	0.00%
	**t8**	80.00%	17.14%	2.86%	0.00%	0.00%
						
**Skin: Pigmentation**	**t1**	100.00%	0.00%	0.00%	0.00%	0.00%
	**t2**	100.00%	0.00%	0.00%	0.00%	0.00%
	**t3**	96.74%	3.26%	0.00%	0.00%	0.00%
	**t4**	95.70%	4.30%	0.00%	0.00%	0.00%
	**t5**	89.13%	10.87%	0.00%	0.00%	0.00%
	**t6**	67.03%	31.87%	1.10%	0.00%	0.00%
	**t8**	80.00%	20.00%	0.00%	0.00%	0.00%
						
**Breast: Symmetry**	**t1**	34.44%	40.00%	22.22%	**3.33%**	0.00%
	**t2**	33.70%	40.22%	22.83%	**3.26%**	0.00%
	**t3**	33.70%	40.22%	22.83%	**3.26%**	0.00%
	**t4**	33.33%	40.86%	22.58%	**3.23%**	0.00%
	**t5**	32.61%	41.30%	22.83%	**3.26%**	0.00%
	**t6**	31.87%	42.86%	23.08%	**2.20%**	0.00%
	**t8**	25.71%	45.71%	25.71%	**2.87%**	0.00%
						
**Breast: Lymphedema**	**t1**	28.89%	66.67%	4.44%	0.00%	0.00%
	**t2**	32.61%	64.13%	3.26%	0.00%	0.00%
	**t3**	29.35%	66.30%	3.26%	**1.09%**	0.00%
	**t4**	22.58%	73.12%	4.30%	0.00%	0.00%
	**t5**	21.74%	73.91%	4.35%	0.00%	0.00%
	**t6**	20.88%	74.73%	4.40%	0.00%	0.00%
	**t8**	25.71%	71.43%	2.86%	0.00%	0.00%
						
**Breast: Pain**	**t1**	84.44%	12.22%	3.33%	0.00%	0.00%
	**t2**	84.78%	14.13%	1.09%	0.00%	0.00%
	**t3**	84.78%	11.96%	3.26%	0.00%	0.00%
	**t4**	83.87%	13.98%	2.15%	0.00%	0.00%
	**t5**	76.09%	21.74%	2.17%	0.00%	0.00%
	**t6**	79.12%	17.58%	3.30%	0.00%	0.00%
	**t8**	91.43%	8.57%	0.00%	0.00%	0.00%

### Longitudinal analyses

**Figure 3:**
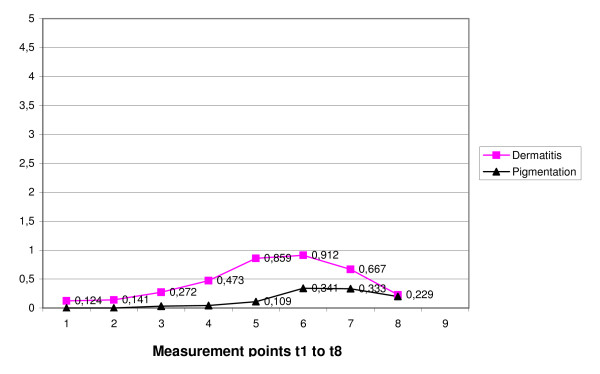
**Temporal variations of scale means of skin toxicity by measurement point (mean grade)**.

Skin toxicity was recorded according "dermatitis" and "pigmentation". Hyperpigmentation was first seen in the third radiation week (mean grade 0.033) and climbed slightly to sixth therapy week (mean grade 0.341). At t8 the mean grade dropped again to 0.20. Dermatitis mean grade increased from the second to last radiation week as well and decreased to t8 (0.229). It was most severe at t6 (mean grade 0.912). Longitudinal skin toxicity variations are detailed in figure 3. Generally, dermatitis was more pronounced than hyperpigmentation.

**Figure 4:**
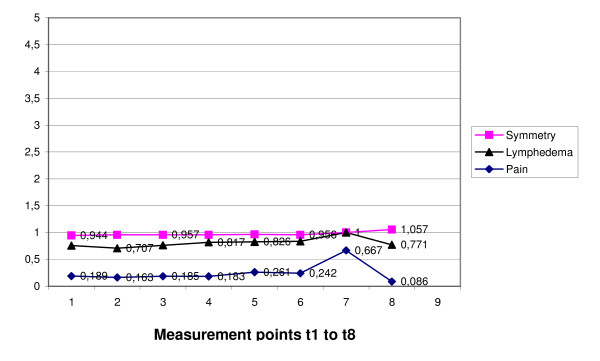
**Temporal variations of scale means of breast toxicity by measurement point (mean grade)**.

Breast toxicity was scored according "symmetry", "lymphedema", and "pain". In the scale "symmetry" a relatively low toxicity level was observed at t1 (postoperative status), which remained constant to t8 (0.944 - 1.057). The scales breast "lymphedema" and "pain" showed the same pattern of longitudinal variations at very low levels. Longitudinal breast toxicity variations are displayed in figure 4. Axillar adverse events were documented according "hair loss", "sweat gland function", and "pain". Toxicity levels in the scales "hair loss" and "sweat gland function" were clinically insignificant (mean grades close to 0). Registered axillar pain was at t1 (postoperative status) low (mean grade 0.57, garde 0 in 51,61%, grade 1 in 39,78%, and grade 2 in 8,60%, grade ≥ grade 3 in 0%) and decreased longitudinally to 0.143 (mean grade at t8). Hair loss and sweat gland function toxicitiy observations revealed very low to 0 levels of toxicity.

For the ipsilateral arm scales "lymphedema", and "function/mobility" were assessed. Arm function was at initial registration (postoperative status/radiotherapy begin) only slightly reduced. However, arm function/mobility improuved continouselly during the course of therapy. Arm lymphedema played a minor role. This data according axilla and ipsilateral arm toxicity is shown in table 3. General symptoms (appetite, nausea, and Karnofsky index) were also recorded and revealed only minor impairments during the complete acute phase of radiotherapy. "Impairments by radiotherapy" which were overall difficulties caused by therapy, showed minor stress with a slight increase at the end of therapy (grade 1 in 37.4%, and grade 2 in 1.1%). However, at t8 (3 months after therapy initiation) only 2.86% of the patients experienced a grade 1 toxicity level and no grade 2 was registered.

**Table 3:** Toxicity documentation: measurement points t1-t8 (Percentages)

Scale	Mesurement-point	Grade 0	Grade 1	Grade 2	Grade 3	Grade 4
**Axilla: Pain**	**t1**	51.61%	39.78%	8.60%	0.00%	0.00%
	**t2**	52.17%	40.22%	7.61%	0.00%	0.00%
	**t3**	53.26%	41.30%	5.43%	0.00%	0.00%
	**t4**	58.06%	38.71%	3.23%	0.00%	0.00%
	**t5**	56.52%	42.39%	1.09%	0.00%	0.00%
	**t6**	63.74%	35.16%	1.10%	0.00%	0.00%
	**t8**	85.71%	14.29%	0.00%	0.00%	0.00%
						
**Axilla: Hair loss**	**t1**	100.00%	0.00%	0.00%	0.00%	xxx
	**t2**	98.91%	1.09%	0.00%	0.00%	xxx
	**t3**	100.00%	0.00%	0.00%	0.00%	xxx
	**t4**	97.85%	1.08%	1.08%	0.00%	xxx
	**t5**	98.91%	1.09%	0.00%	0.00%	xxx
	**t6**	100.00%	0.00%	0.00%	0.00%	xxx
	**t8**	100.00%	0.00%	0.00%	0.00%	xxx
						
**Axilla: sweat gland function**	**t1**	100.00%	0.00%	0.00%	0.00%	xxx
	**t2**	100.00%	0.00%	0.00%	0.00%	xxx
	**t3**	100.00%	0.00%	0.00%	0.00%	xxx
	**t4**	100.00%	0.00%	0.00%	0.00%	xxx
	**t5**	100.00%	0.00%	0.00%	0.00%	xxx
	**t6**	100.00%	0.00%	0.00%	0.00%	xxx
	**t8**	100.00%	0.00%	0.00%	0.00%	xxx
						
**Arm: Lymphedema**	**t1**	62.37%	37.63%	0.00%	0.00%	0.00%
	**t2**	60.87%	38.04%	1.09%	0.00%	0.00%
	**t3**	63.04%	36.96%	0.00%	0.00%	0.00%
	**t4**	61.29%	38.71%	0.00%	0.00%	0.00%
	**t5**	61.96%	38.04%	0.00%	0.00%	0.00%
	**t6**	62.64%	37.36%	0.00%	0.00%	0.00%
	**t8**	60.00%	37.14%	2.86%	0.00%	0.00%
						
**Arm: Function**	**t1**	39.78%	53.76%	6.45%	0.00%	0.00%
	**t2**	42.39%	54.35%	3.26%	0.00%	0.00%
	**t3**	46.15%	52.75%	1.10%	0.00%	0.00%
	**t4**	48.39%	51.61%	0.00%	0.00%	0.00%
	**t5**	53.26%	46.74%	0.00%	0.00%	0.00%
	**t6**	59.34%	39.56%	1.10%	0.00%	0.00%
	**t8**	71.43%	28.57%	0.00%	0.00%	0.00%

### Univariate analyses by age

Univariate analyses revealed slightly higher toxicity in older (> 60 years) versus young patients (< 50 years) in 2 toxicity scales: breast-symmetry (p = 0.033), and arm function (p = 0.007). However, in the scale "appetite" registred toxicity was higher in younger (< 50 years) versus older (> 60 years) patients (p = 0.039). Toxicity differences in all other scales were not significant. Between older (> 60 years) and midaged patients (50-60 years) no statistically significant differences in toxicity could be detected. This was also true for the comparison between young (< 50 years) versus midaged patient groups (50-60 years).

## Discussion

The present study addresses by using a prospective design specifically the radiotherapy toxicity in 109 patients during the acute phase (day 0 to day 90 from radiotherapy initiation) of postoperative breast 3D conformal irradiation following breast-conserving surgery. Mean age in the analyzed study cohort was 55 years. Toxicity assessment and documentation modeled according to the international CTC classification for acute toxicity in oncology [10] using new developed instruments [11,12]. Classical CTC criteria were supplemented by radiotherapy specific aspects. Longitudinal analyses showed, as expected, a slight increase in skin radiotherapy reactions (pigmentation and dermatitis) during the course of irradiation. However, the continuous decrease of axillar pain and arm dysfunction registering the highest level of toxicity at therapy initiation was rather surprising. Surprisingly was also that the postoperative level of physical deficits at radiotherapy begin (t1) was relatively low, and this level even decreased during postoperative radiotherapy. Generally, the registered overall mean grade of toxicity was very low: 0 to 1.057, which supports the breast- conserving therapy as a treatment entity consisting of surgery, irradiation, and systemic treatment. Literature reports of acute toxicity for radiotherapy after breast-conserving surgery are infrequent. This endpoint is historically neglected, although acute toxicity is discriminating against good or poor compliance, and this is especially true by stratifying to the variable "age". Vicini et al. [19] reported 281 patients treated with intensity modulated 3D radiotherapy. No patient showed skin toxicity higher than grade 3. Grade 0 or 1 toxicity was registered in 157 (56%), grade 2 in 102 patients (43%), and grade 3 in only three women (1%). This was in concordance to our results with only 1.1% grade 3-dermatitis at measurement point t 5. Grade 1 and 2 skin reactions were registered in our study population highest at the end of radiation (t 6) with 51.65% and 19.78%, respectively.

Gruber and coworkers could show that the expansion of treated volume to the locoregional lymphnodes for patients with extranodal tumor invasion and/or other negative prognostic factors provides a sufficient compensation for better survival [20]. In our study population 22% of the patients were irradiated also in the supraclavicular region, and 12.8% in both axillar and supraclavicular areas. However, toxicity levels according axilla and arm scales were extremely low. In fact, axillar pain did improve from initial level of 39.8% grade 1 and 8.6% grade 2 at measurement point t1 to 14.3% grade 1 and 0% grade 2 at measurement point t8. Arm function showed the same favorable kinetics from 53.8% grade 1 and 6.4% grade 2 at measurement point t1 to 28.6% grade 1 and 0% grade 2 at measurement point t8. These findings support the assumption that the postoperative healing process in the axilla and the ipsilateral arm is not affected or significantly disturbed by moderate doses of radiotherapy of 46 Gy. Arm lymphedema was, however, consistently observed at low levels throughout of the entire study observation period of three months with no improving or deteriorating temporal trend. Toxicities grade 3 or higher were not observed in this regard. Albrecht et al. [21] compared 129 patients with axillar radiotherapy after breast-conserving surgery versus 173 patients after breast-conserving surgery and axillar dissection. Arm lymphedema, axillar pain and arm function restrictions were observed in 26% of women with axilla surgery, but in only 1% following axillar radiotherapy. These data confirm our results of low toxicity following locoregional radiation therapy in addition to breast-conserving surgery. At measurement point t8 (6 weeks after radiotherapy) 97.1% of the present study patients had no impairments from radiotherapy at all and only 2.8% stated very low general restrictions or burdens due to radiotherapy. Longitudinally only 1.1% of the women complained at measurement point t6 moderate stress (grade 2) in the course of radiotherapy. Nagel and coworkers [22] confirmed in a field study with breast cancer patients a high rate of adjuvant radiotherapy in addition to breast-conserving surgery of 90.6%. The study documented a very impressive high level of acceptance of adjuvant radiotherapy after breast-conserving surgical care which was in concordance with our results. However, univariate analyses in this trial revealed higher age and co-morbidity as negative prognostic variables for use of radiotherapy. This finding did not correspond to our study results. We could not demonstrate a significant difference in toxicity discriminating between the different age groups: <50 years versus 50-60 versus >60 years. However, we admit limitations in terms of cohort sample size, and considering chronological age and not the biological age.

## Conclusion

These prospective measurement results of toxicity according CTC-classification during postoperative adjuvant 3D radiotherapy following breast-conserving surgery demonstrated very low side-effect levels throughout the entire acute treatment phase. Thus, the BCT concept for breast cancer was generally very well tolerated. On the contrary, postoperative radiation did not impaired recovery from surgery. Axillar pain and arm dysfunction improved continuously during irradiation course. Toxicity-measurement with the new topographic module was feasible. Univariate analyses by age could not reveal clinical meaningful differences between the assessed young and older study cohorts. In consequence, not modified standard treatments for breast cancer should be performed in elderly population as well. Further longitudinal data is needed to assess temporal outcome variations at long-term follow-up.

## Competing interests

The authors declare that they have no competing interests.

## Authors' contributions

RG developed and clinically introduced at the institution the technique of CT-based, 3D conformal radiation following breast-conserving surgery in the late 1990 s. RG also developed the present study concept of creating a comprehensive data base for long-term temporal analyses of 3D conformal adjuvant radiotherapy in terms of survivor outcomes, conducted the study and was activelly participating in data collection as well. JS participated in the technical development, in the data collection and drafted the manuscript. JS was also involved in the clinical patient management. KE participated in the study development especially by helping to define the study endpoints. KE was also actively involved in the data collection. BK helped to define the principle concept of the study as a long-term institutional goal. BK also participated in study design and its coordination. All authors read and approved the final manuscript.
